# Laparoscopic versus open liver resection in hepatocellular carcinoma patients with sarcopenia: a retrospective cohort study

**DOI:** 10.3389/fnut.2026.1821779

**Published:** 2026-09-30

**Authors:** Jingwei Zheng, Jiani Lu, Jinnuo He, Wenjun Zheng, Wentao Cai

**Affiliations:** 1 Department of Gastrointestinal Surgery, The First Affiliated Hospital of Wenzhou Medical University, Wenzhou, China; 2 Trauma & Emergency Surgery, The First Affiliated Hospital of Wenzhou Medical University, Wenzhou, China

**Keywords:** body composition, carcinoma, hepatocellular, hepatectomy, laparoscopy, sarcopenia

## Abstract

**Background:**

Sarcopenia identifies patients with hepatocellular carcinoma (HCC) who have reduced physiological reserve and an increased risk of adverse outcomes after hepatectomy. However, whether laparoscopic liver resection (LLR) is associated with better perioperative and long-term outcomes than open liver resection (OLR) in this vulnerable population remains uncertain.

**Methods:**

We retrospectively evaluated consecutive adults with pathologically confirmed HCC and preoperative computed tomography-defined sarcopenia who underwent curative-intent liver resection at a high-volume center in China between January 2017 and December 2021. The primary outcomes were recurrence-free survival (RFS) and overall survival (OS). One-to-one propensity score matching was used as the primary adjustment method, with overlap weighting and stabilized inverse probability of treatment weighting as sensitivity analyses.

**Results:**

Among 285 patients, 110 underwent LLR and 175 underwent OLR. Compared with OLR, LLR was associated with lower estimated blood loss (median, 150 vs. 200 mL), a shorter postoperative hospital stay (12 vs. 13 days), and fewer Clavien–Dindo grade ≥II complications (20.9% vs. 34.3%), whereas major complications, anatomical resection rates, and wide resection margins were comparable. After propensity score matching, 96 patients were retained in each group. During a median follow-up of approximately 55 months, LLR was associated with longer RFS (HR 0.56, 95% CI 0.37–0.84) and OS (HR 0.54, 95% CI 0.34–0.87). Similar associations were observed in the overlap-weighted and stabilized inverse probability-weighted analyses.

**Conclusion:**

Among selected HCC patients with computed tomography-defined sarcopenia, LLR was associated with a lower perioperative burden and favorable long-term outcomes without an apparent compromise in major morbidity or technical oncologic quality. Prospective multicenter studies are required to confirm these findings and determine whether reduced surgical stress contributes to preservation of muscle health and functional recovery.

## Introduction

Hepatocellular carcinoma (HCC) remains a major global health burden and a leading cause of cancer-related mortality (1). Curative-intent therapies, including liver resection, offer meaningful survival benefits for selected patients with early-stage disease and preserved liver function (2, 3). Nevertheless, tumor recurrence remains a critical challenge after hepatectomy, with reported 5-year recurrence risks approaching 50–70% even after curative resection (4), underscoring the need to optimize perioperative management and surgical strategies without compromising oncologic principles.

Sarcopenia, characterized by the progressive loss of skeletal muscle mass and function, is increasingly recognized as a clinically relevant host factor in patients with chronic liver disease and HCC (5). Systematic reviews and meta-analyses have shown that sarcopenia is common in HCC and is associated with inferior survival outcomes (6). Although consensus definitions of sarcopenia emphasize both muscle quantity and muscle function, CT-based body composition assessment at the third lumbar vertebra (L3) provides a practical and reproducible method to quantify skeletal muscle mass in routine oncology care, enabling risk stratification in retrospective surgical cohorts (7).

Laparoscopic liver resection (LLR) has evolved substantially over the past two decades, supported by international consensus statements and guidelines (8). Compared with open liver resection (OLR), LLR has been associated with less surgical trauma and improved perioperative recovery in many observational studies, while maintaining oncologic adequacy in appropriately selected patients (9). Furthermore, multicenter propensity score analyses have expanded the evidence base across clinically relevant HCC subgroups, such as early-stage disease with or without microvascular invasion and patients with metabolic comorbidities (10).

However, whether the potential benefits of LLR extend to HCC patients with CT-defined sarcopenia remains insufficiently defined. These patients may be particularly vulnerable to surgical stress and postoperative functional decline. Emerging data suggest that LLR may be associated with less postoperative skeletal muscle loss than the open approach, providing a possible clinical rationale for evaluating minimally invasive surgery in this vulnerable population (11). Therefore, we conducted a retrospective cohort study at a high-volume hepatobiliary center in China to compare perioperative and long-term outcomes of LLR versus OLR among HCC patients with CT-defined preoperative sarcopenia, using propensity score-based methods to reduce confounding by indication.

## Methods

### Study design and patient population

This retrospective cohort study evaluated perioperative and long-term outcomes of LLR versus OLR in patients with HCC and preoperative CT-defined sarcopenia. Consecutive adult patients who underwent curative-intent liver resection between January 2017 and December 2021 at the First Affiliated Hospital of Wenzhou Medical University were screened.

Patients were eligible if they (1) had pathologically confirmed HCC on the resection specimen, (2) underwent hepatectomy with curative intent, and (3) had a preoperative abdominal computed tomography (CT) scan suitable for body composition assessment. Patients were excluded according to prespecified criteria, including non-HCC pathology, prior non-surgical antitumor treatment before hepatectomy, non-curative resection, R2 resection, palliative surgery, macrovascular invasion, unavailable or non-evaluable preoperative CT for L3 skeletal muscle measurement, CT performed outside the prespecified time window, incomplete key follow-up data, and duplicate records or repeated admissions for the same patient. Exclusion criteria were applied hierarchically, and each patient was counted only once in the selection flowchart. For patients with duplicate records or repeated admissions, only the index hepatectomy record was retained. Patients with missing key eligibility information, unavailable or non-evaluable CT images, CT scans outside the prespecified time window, or incomplete key follow-up data were excluded from the analytic cohort. For baseline covariates included in propensity score estimation and Cox regression analyses, complete-case analysis was performed, and no imputation was used.

The study protocol was approved by the Ethics Committee of the First Affiliated Hospital of Wenzhou Medical University. The requirement for informed consent was waived due to the retrospective nature of the study.

### Exposure definition and surgical approach

The exposure of interest was surgical approach, categorized as LLR or OLR based on operative records. Conversion from laparoscopy to open surgery was permitted when clinically indicated and was recorded descriptively. Patients who underwent conversion were analyzed in the OLR group according to the as-treated classification. Operative characteristics were abstracted from operative notes, including resection type, resection extent, anatomical resection, use of inflow occlusion with the Pringle maneuver, duration of inflow occlusion, operative time, estimated blood loss, perioperative transfusion, and surgical margin status.

### CT-based definition of sarcopenia

In this retrospective surgical oncology cohort, sarcopenia was operationally defined as CT-defined low skeletal muscle mass using preoperative CT-derived L3 skeletal muscle index (SMI), according to sex-specific cutoff values established in a previous study (12). Preoperative CT scans performed within 7 days before surgery were used for assessment. Skeletal muscle area was quantified on a single axial CT slice at the L3. Muscle segmentation was performed using ImageJ with a predefined Hounsfield unit threshold for skeletal muscle of −29 to 150 HU. SMI was calculated as skeletal muscle area (cm^2^) divided by height squared (m^2^). Low SMI was defined as <51 cm^2^/m^2^ for male patients and <37 cm^2^/m^2^ for female patients.

### Outcomes and follow-up

The primary long-term outcomes were recurrence-free survival (RFS) and overall survival (OS). Time zero was the date of hepatectomy. RFS was analyzed as a composite endpoint, defined as time to first documented tumor recurrence or death from any cause, whichever occurred first. OS was defined as time to death from any cause. Patients without an event were censored at the date of last follow-up. Follow-up was administratively censored on January 1, 2026.

Short-term outcomes included postoperative length of stay and postoperative complications graded using the Clavien-Dindo classification. Complications were analyzed as grade ≥II, and major complications were analyzed as grade ≥III.

### Covariates

Clinical, laboratory, radiological, pathological, operative, and treatment-related variables were extracted from the medical records. Baseline covariates for propensity score estimation were prespecified based on clinical knowledge and a directed acyclic graph (DAG) representing the assumed causal relationships between surgical approach and long-term outcomes (Supplementary Figure S1). These covariates included demographics (age and sex), body composition (body mass index), liver disease status and liver function (hepatitis B virus infection, hepatitis B virus DNA, cirrhosis, Child-Pugh class, and albumin-bilirubin grade), tumor burden and stage (maximum tumor diameter, tumor number, and Barcelona Clinic Liver Cancer stage), and baseline laboratory and biomarker measures (white blood cell count, hemoglobin, platelet count, alanine aminotransferase, aspartate aminotransferase, gamma-glutamyl transferase, alkaline phosphatase, and alpha-fetoprotein).

Additional preoperative tumor characteristics, including tumor location, were also recorded and reported descriptively. Pathological, operative, and treatment-related variables were collected where available, including microvascular invasion, tumor differentiation, satellite nodules, anatomical resection, surgical margin, postoperative adjuvant therapy, and post-recurrence treatment strategies. Intraoperative and postoperative variables were not included in the primary propensity score model.

### Statistical analysis

All analyses were performed in R (version 4.5.0). Continuous variables are presented as median (interquartile range) and compared using the Mann–Whitney *U* test. Categorical variables are presented as counts (percentages) and compared using the chi-square test or Fisher’s exact test.

To address confounding by indication, propensity scores for receiving LLR were estimated using multivariable logistic regression with prespecified baseline covariates. The primary adjusted analysis used 1:1 nearest-neighbor PSM without replacement with a caliper width of 0.2 standard deviations of the logit of the propensity score. Two additional propensity score-based weighting approaches were conducted as sensitivity analyses: stabilized inverse probability of treatment weighting (sIPTW) and overlap weighting targeting the average treatment effect in the overlap population (ATO). For the sIPTW analysis, extreme weights were truncated at the 1st and 99th percentiles. Overlap weights were used without additional truncation because they are intrinsically bounded. Covariate balance was assessed using absolute standardized mean differences (SMDs).

OS and RFS were estimated using the Kaplan–Meier method and compared using the log-rank test. Hazard ratios (HRs) with 95% confidence intervals (CIs) were estimated using Cox proportional hazards models. Robust variance estimation was applied for matched and weighted analyses. Conventional Cox regression analyses were first performed in the overall cohort. As a sensitivity analysis, expanded multivariable Cox regression models were fitted in the overall cohort by incorporating available tumor location, pathological, surgical, and treatment-related variables, including microvascular invasion, tumor differentiation, satellite nodules, surgical resection type, surgical margin, and postoperative adjuvant therapy. Prespecified subgroup analyses were performed in the propensity score-matched cohort, and effect modification was assessed using interaction terms. All statistical tests were two-sided, and *p* values <0.05 were considered statistically significant.

## Results

### Patient selection and study cohort

From January 2017 to December 2021, 2,367 consecutive patients who underwent curative-intent liver resection were screened (Figure 1). Patients were excluded because of non-HCC pathology (*n* = 635), previous non-surgical antitumor treatment (*n* = 383), missing or non-evaluable preoperative CT for L3 muscle measurement (*n* = 274), CT performed outside the prespecified time window (*n* = 57), macrovascular invasion (*n* = 144), non-curative, R2, or palliative resection (*n* = 122), missing key follow-up data (*n* = 61), duplicate records or repeated admissions (*n* = 39), or age <18 years (*n* = 3). After applying these criteria, 649 eligible HCC patients with evaluable preoperative CT remained, of whom 364 did not meet the CT-based definition of sarcopenia. The final analytic cohort comprised 285 HCC patients with CT-defined sarcopenia, including 110 who underwent LLR and 175 who underwent OLR (Figure 1).

**Figure 1:**
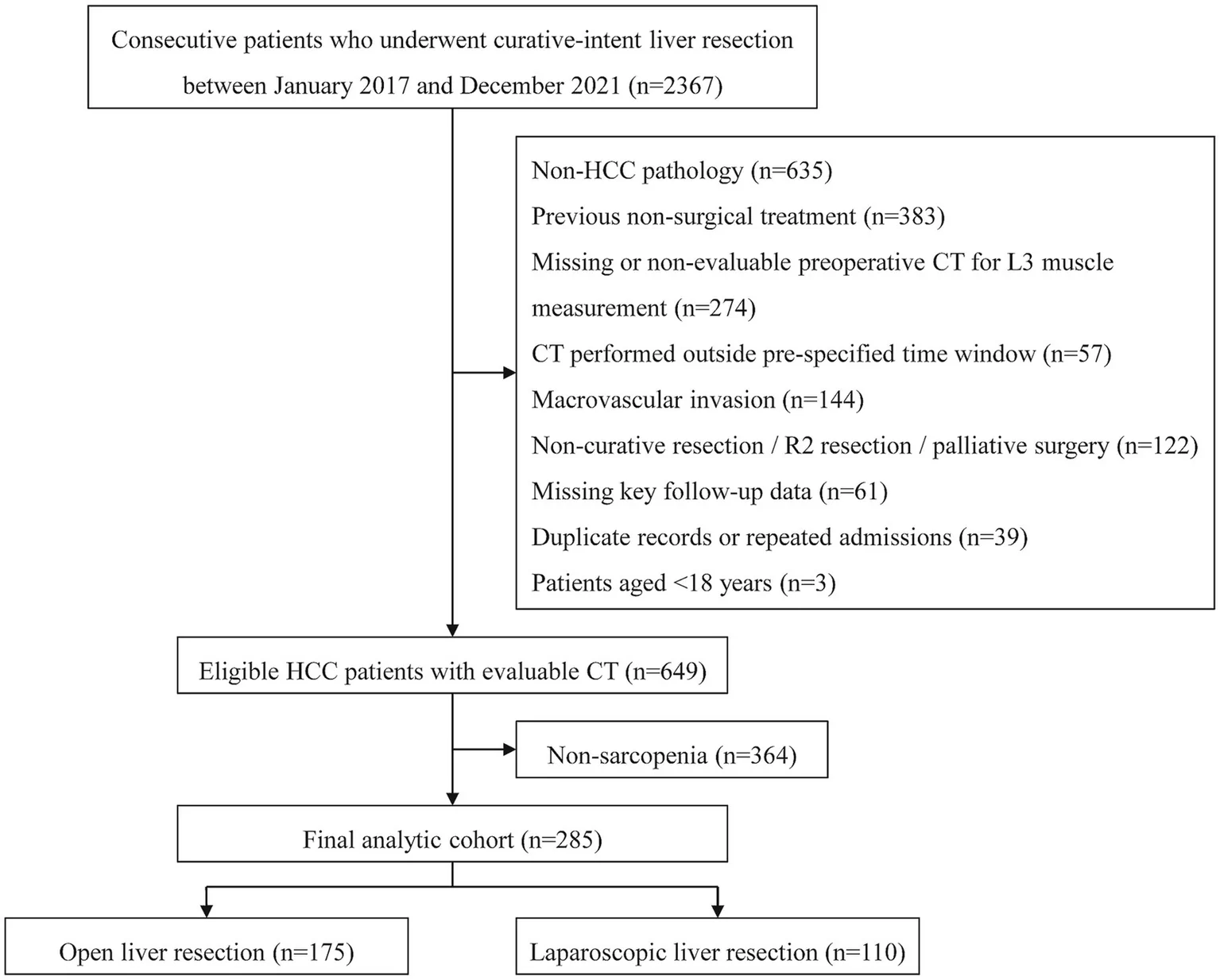
Patient selection flowchart. Flow diagram showing screening, exclusions, eligibility assessment, and final cohort allocation to laparoscopic liver resection (LLR) and open liver resection (OLR) from January 2017 to December 2021.

Baseline characteristics before and after PSM are summarized in Table 1. Before matching, the LLR and OLR groups were broadly comparable in age, sex, body mass index (BMI), hepatitis B virus (HBV)-related variables, liver function variables, most laboratory parameters, microvascular invasion (MVI), tumor differentiation, satellite nodules, and postoperative adjuvant therapy. However, imbalances were observed for albumin-bilirubin (ALBI) grade (*p* = 0.027), tumor location (central location, 32.7% vs. 46.3%, *p* = 0.024), and maximum tumor diameter >5 cm (47.3% vs. 60.0%, *p* = 0.035), with a higher proportion of central tumors and larger tumors in the OLR group. After 1:1 PSM, 96 patients in each group were retained, and baseline balance improved across most measured covariates.

**Table 1:** Baseline characteristics of the study cohort.

	Before PSM			After PSM		
Characteristic	LLR (*n* = 110)	OLR (*n* = 175)	*p* value	LLR (*n* = 96)	OLR (*n* = 96)	*p* value
Age, ≥65 years, *n* (%)	21 (19.1%)	31 (17.7%)	0.770	19 (19.8%)	20 (20.8%)	0.858
Sex, male, *n* (%)	83 (75.5%)	147 (84.0%)	0.075	75 (78.1%)	76 (79.2%)	0.860
BMI, ≥24, *n* (%)	64 (58.2%)	102 (58.3%)	0.986	57 (59.4%)	55 (57.3%)	0.770
Current smoking history, *n* (%)	42 (38.2%)	76 (43.4%)	0.381	40 (41.7%)	36 (37.5%)	0.555
Heavy alcohol consumption history, *n* (%)	35 (31.8%)	68 (38.9%)	0.229	33 (34.4%)	31 (32.3%)	0.759
WBC, <10 × 10^9^/L, *n* (%)	6 (5.5%)	4 (2.3%)	0.192	3 (3.1%)	3 (3.1%)	1.000
Hb, <120 g/L, *n* (%)	11 (10.0%)	13 (7.4%)	0.447	8 (8.3%)	9 (9.4%)	0.799
PLT, <100 × 10^9^/L, *n* (%)	22 (20.0%)	28 (16.0%)	0.387	20 (20.8%)	18 (18.8%)	0.717
ALT, ≥40 U/L, *n* (%)	34 (30.9%)	63 (36.0%)	0.377	28 (29.2%)	29 (30.2%)	0.874
AST, ≥40 U/L, *n* (%)	33 (30.0%)	58 (33.1%)	0.580	27 (28.1%)	29 (30.2%)	0.751
GGT, ≥50 U/L, *n* (%)	47 (42.7%)	86 (49.1%)	0.291	39 (40.6%)	41 (42.7%)	0.770
ALP, ≥130 U/L, *n* (%)	7 (6.4%)	22 (12.6%)	0.091	4 (4.2%)	6 (6.3%)	0.516
HBV DNA, ≥2000 IU/mL, *n* (%)	41 (37.3%)	62 (35.4%)	0.752	36 (37.5%)	31 (32.3%)	0.449
AFP, ≥400 ug/L, *n* (%)	53 (48.2%)	94 (53.7%)	0.363	44 (45.8%)	43 (44.8%)	0.885
HBV infection, *n* (%)	85 (77.3%)	141 (80.6%)	0.503	75 (78.1%)	73 (76.0%)	0.731
Child Pugh class B, *n* (%)	3 (2.7%)	1 (0.6%)	0.302	0	0	1.000
ALBI grade, *n* (%)			0.027			0.505
1	76 (69.1%)	141 (80.6%)		70 (72.9%)	74 (77.1%)	
2	34 (30.9%)	34 (19.4%)		26 (27.1%)	22 (22.9%)	
Cirrhosis, *n* (%)	67 (60.9%)	116 (66.3%)	0.357	60 (62.5%)	61 (63.5%)	0.881
Tumor location, central, *n* (%)	36 (32.7%)	81 (46.3%)	0.024	29 (30.2%)	42 (43.8%)	0.052
Maximum tumor diameter, >5 cm, *n* (%)	52 (47.3%)	105 (60.0%)	0.035	46 (47.9%)	44 (45.8%)	0.772
Tumor number, Multiple, *n* (%)	2 (1.8%)	6 (3.4%)	0.715	1 (1.0%)	0 (0.0%)	1.000
BCLC stage, *n* (%)			0.207			0.650
0	14 (12.7%)	12 (6.9%)		12 (12.5%)	10 (10.4%)	
A	95 (86.4%)	159 (90.9%)		84 (87.5%)	86 (89.6%)	
B	1 (0.9%)	4 (2.3%)		0	0	
MVI, *n* (%)	56 (50.9%)	94 (53.7%)	0.644	48 (50.0%)	45 (46.9%)	0.665
Differentiation, III–IV, *n* (%)	55 (50.0%)	83 (47.4%)	0.672	47 (49.0%)	43 (44.8%)	0.563
Satellite, *n* (%)	17 (15.5%)	34 (19.4%)	0.394	14 (14.6%)	18 (18.8%)	0.439
Adjuvant therapy, *n* (%)	21 (19.1%)	38 (21.7%)	0.595	18 (18.8%)	15 (15.6%)	0.566

AFP, alpha-fetoprotein; ALBI, albumin–bilirubin; ALP, alkaline phosphatase; ALT, alanine aminotransferase; AST, aspartate aminotransferase; BCLC, Barcelona Clinic Liver Cancer; BMI, body-mass index; GGT, gamma-glutamyl transferase; Hb, hemoglobin; HBV, hepatitis B virus; LLR laparoscopic liver resection; OLR open liver resection; PLT, platelet count; PSM, propensity score matching; WBC, white blood cell count.

### Propensity score diagnostics

Propensity score distributions and covariate balance diagnostics are shown in Figures 2A,B. After PSM, the distribution of propensity scores overlapped substantially between groups, and absolute SMDs decreased across prespecified baseline covariates, indicating improved balance after matching.

**Figure 2:**
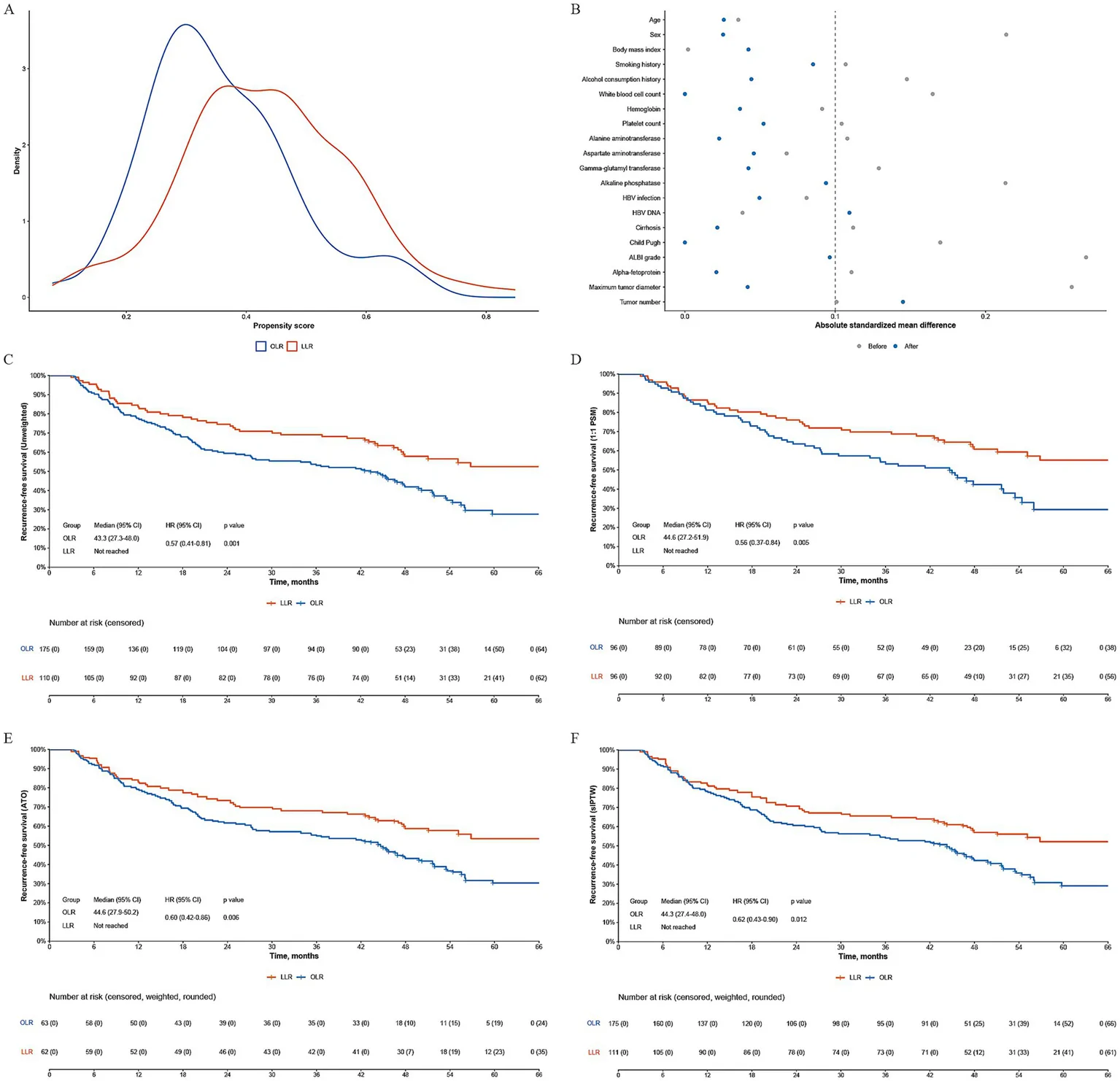
Propensity score diagnostics and recurrence-free survival analyses. **(A)** Distribution of propensity scores by surgical approach. **(B)** Covariate balance before and after propensity score matching, shown as absolute standardized mean differences. **(C–F)** Kaplan–Meier curves for recurrence-free survival comparing laparoscopic liver resection (LLR) and open liver resection (OLR) under different analytic approaches: **(C)** unweighted (crude) analysis, **(D)** propensity score matching (PSM), **(E)** overlap weighting targeting the average treatment effect in the overlap population (ATO), and **(F)** stabilized inverse probability of treatment weighting (sIPTW). ATO, average treatment effect in the overlap population; LLR, laparoscopic liver resection; OLR, open liver resection; PSM, propensity score matching; sIPTW, stabilized inverse probability of treatment weighting.

### Intraoperative and postoperative short-term outcomes

Intraoperative and short-term postoperative outcomes are presented in Table 2. Rates of anatomical resection and wide resection margin (≥1 cm) were comparable between the LLR and OLR groups (20.0% vs. 18.3%, *p* = 0.719; and 56.4% vs. 58.3%, *p* = 0.749, respectively). Use of the Pringle maneuver and the duration of inflow occlusion were also similar between groups. Conversion from laparoscopy to open surgery occurred in 2 patients and was analyzed in the OLR group according to the as-treated classification.

**Table 2:** Intraoperative and postoperative short-term results.

Characteristic	LLR (*n* = 110)	OLR (*n* = 175)	*p* value
Anatomical resection, *n* (%)	22 (20.0%)	32 (18.3%)	0.719
Wide resection margin (≥1 cm), *n* (%)	62 (56.4%)	102 (58.3%)	0.749
Pringle maneuver, *n* (%)	85 (77.3%)	126 (72.0%)	0.323
Duration of occluding (min), median (Q1, Q3)	17.0 (13.0, 20.0)	16.0 (12.0, 20.0)	0.217
Blood loss (mL), median (Q1, Q3)	150 (50, 250)	200 (100, 250)	<0.001
Transfusion, *n* (%)	8 (7.3%)	17 (9.7%)	0.478
Operative time (min), median (Q1, Q3)	190 (157, 225)	188 (159, 221)	0.767
Hospitalization (day), median (Q1, Q3)	12.00 (10.00, 14.00)	13.00 (11.00, 15.00)	0.005
Clavien-Dindo classification ≥ II, *n* (%)	23 (20.9%)	60 (34.3%)	0.016
Clavien-Dindo classification ≥ III, *n* (%)	4 (3.6%)	12 (6.9%)	0.250
Converted from laparoscopy to open surgery, *n* (%)		2 (1.8%)	

LLR, laparoscopic liver resection; OLR, open liver resection.

LLR was associated with lower estimated blood loss (median 150 mL vs. 200 mL, *p* < 0.001) and shorter postoperative length of stay (median 12 vs. 13 days, *p* = 0.005). Transfusion rate and operative time did not differ significantly between groups. Postoperative complications graded as Clavien-Dindo ≥II occurred less frequently after LLR than after OLR (20.9% vs. 34.3%, *p* = 0.016), whereas major complications (Clavien-Dindo ≥III) were comparable between groups (3.6% vs. 6.9%, *p* = 0.250) (Figure 3).

**Figure 3:**
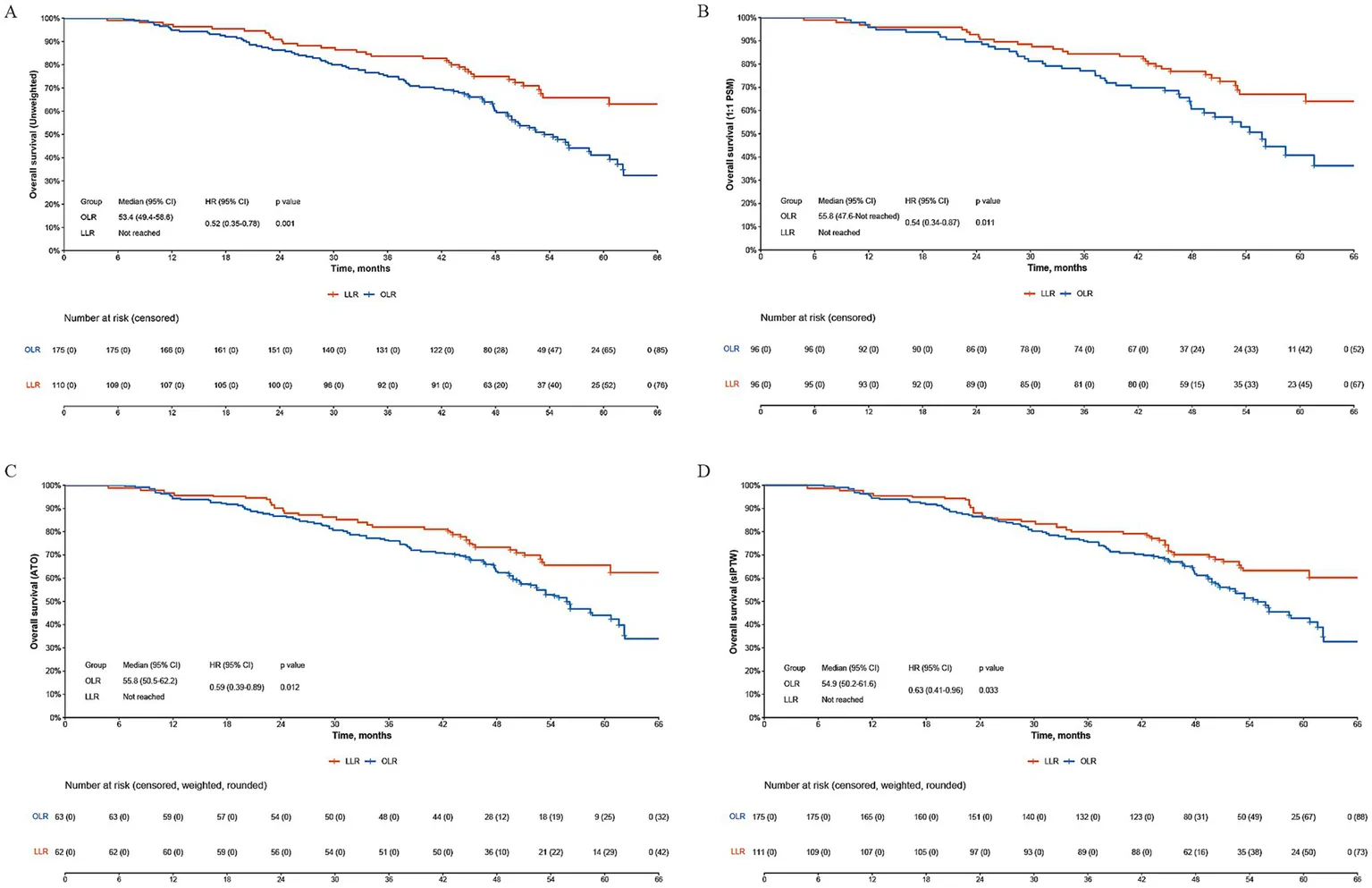
Overall survival analyses by analytic approach. Kaplan–Meier curves for overall survival comparing laparoscopic liver resection (LLR) and open liver resection (OLR) under different analytic approaches: **(A)** Unweighted (crude) analysis, **(B)** Propensity score matching (PSM), **(C)** Overlap weighting targeting the average treatment effect in the overlap population (ATO), and **(D)** Stabilized inverse probability of treatment weighting (sIPTW). ATO, average treatment effect in the overlap population; LLR, laparoscopic liver resection; OLR, open liver resection; PSM, propensity score matching; sIPTW, stabilized inverse probability of treatment weighting.

### Long-term outcomes

The median follow-up was 54.9 months (95% CI, 51.6–57.8) in the LLR group and 55.1 months (95% CI, 52.4–58.1) in the OLR group, with follow-up censored on January 1, 2026. During follow-up, RFS events occurred in 48 of 110 patients in the LLR group and 111 of 175 patients in the OLR group. Deaths occurred in 34 of 110 patients in the LLR group and 90 of 175 patients in the OLR group.

For RFS, the unweighted analysis showed a median RFS of 43.3 months (95% CI, 27.3–48.0) in the OLR group and not reached in the LLR group, with a HR for LLR versus OLR of 0.57 (95% CI, 0.41–0.81; *p* = 0.001) (Figure 2C). In the PSM cohort, the median RFS was 44.6 months (95% CI, 27.2–51.9) for OLR and not reached for LLR (HR 0.56, 95% CI, 0.37–0.84; *p* = 0.005) (Figure 2D). Results were consistent in the overlap weighting analysis targeting the average treatment effect in the ATO, with a median RFS of 44.6 months (95% CI, 27.9–50.2) for OLR and not reached for LLR (HR 0.60, 95% CI, 0.42–0.86; *p* = 0.006) (Figure 2E), and in the sIPTW analysis, with a median RFS of 44.3 months (95% CI, 27.4–48.0) for OLR and not reached for LLR (HR 0.62, 95% CI, 0.43–0.90; *p* = 0.012) (Figure 2F).

Consistent results were observed in Cox regression analyses. In the overall cohort, LLR was associated with improved RFS in univariable analysis (HR 0.57, 95% CI, 0.41–0.81; *p* = 0.001), and the association remained significant after expanded multivariable adjustment incorporating available tumor location, pathological, surgical, and treatment-related variables (adjusted HR 0.63, 95% CI, 0.44–0.88; *p* = 0.008) (Supplementary Table S1).

For overall survival (OS), the unweighted analysis showed a median OS of 53.4 months (95% CI, 49.4–58.6) in the OLR group and not reached in the LLR group (HR 0.52, 95% CI, 0.35–0.78; *p* = 0.001). In the PSM cohort, the median OS was 55.8 months (95% CI, 47.9–not reached) for OLR and not reached for LLR (HR 0.54, 95% CI, 0.34–0.87; *p* = 0.011). Findings were similar in the ATO analysis (median OS 55.8 months, 95% CI, 50.5–62.2 for OLR vs. not reached for LLR; HR 0.59, 95% CI, 0.39–0.89; *p* = 0.012) and in the sIPTW analysis (median OS 54.9 months, 95% CI, 50.2–61.6 for OLR vs. not reached for LLR; HR 0.63, 95% CI, 0.41–0.96; *p* = 0.033).

Similarly, Cox regression analyses supported an OS advantage for LLR. Univariable analysis showed a lower risk of death with LLR versus OLR (HR 0.52, 95% CI, 0.35–0.78; *p* = 0.001), which persisted after expanded multivariable adjustment incorporating available tumor location, pathological, surgical, and treatment-related variables (adjusted HR 0.62, 95% CI, 0.41–0.93; *p* = 0.021) (Supplementary Table S2).

### Recurrence patterns and post-recurrence treatment

Recurrence-related characteristics and post-recurrence treatment strategies in the PSM cohort are summarized in Supplementary Table S3. The distribution of first RFS event type was comparable between the LLR and OLR groups, including intrahepatic recurrence, extrahepatic recurrence, combined intrahepatic and extrahepatic recurrence, and death without documented recurrence (*p* = 0.681). The proportions of early recurrence within 2 years and late recurrence after 2 years were also similar between groups (*p* = 0.857). Among patients with documented recurrence, post-recurrence treatment strategies, including repeat resection, ablation, transarterial chemoembolization/hepatic arterial infusion chemotherapy (TACE/HAIC), systemic therapy, radiotherapy, and best supportive care, were broadly comparable between the LLR and OLR groups (*p* = 0.778).

### Subgroup analyses

Prespecified subgroup analyses in the PSM cohort are shown in Supplementary Figure S2. Hazard ratios for LLR versus OLR were estimated for both RFS (Supplementary Figure S2A) and OS (Supplementary Figure S2B) across strata defined by age, sex, BMI, HBV infection, cirrhosis, ALBI grade, alpha-fetoprotein (AFP), and Barcelona Clinic Liver Cancer (BCLC) stage. Interaction testing did not suggest substantial heterogeneity for most subgroup variables. For OS, an exploratory interaction signal was observed by BMI category (*p* for interaction = 0.041; Supplementary Figure S2B), whereas interaction tests for RFS were not statistically significant across the prespecified subgroups (Supplementary Figure S2A). This finding should be interpreted cautiously given the limited sample size and multiple subgroup comparisons.

## Discussion

In this retrospective cohort of HCC patients with CT-defined sarcopenia, laparoscopic liver resection was associated with a lower perioperative burden and more favorable long-term outcomes compared with open liver resection. A key aspect of our findings is that the short-term advantages of LLR were observed while surgical and oncologic quality indicators, including anatomical resection rate and wide resection margin, were comparable between groups. This suggests that, in appropriately selected patients, the minimally invasive approach did not appear to compromise operative intent or technical oncologic adequacy.

Perioperatively, LLR was associated with lower estimated blood loss, a shorter postoperative hospital stay, and fewer Clavien–Dindo grade ≥II complications, whereas major morbidity was comparable between the two groups. Although the absolute difference in postoperative hospital stay was modest, this finding should not be interpreted in isolation. Rather, the shorter hospital stay, lower blood loss, and lower incidence of clinically relevant complications collectively suggest a reduction in perioperative burden. This may be particularly relevant in patients with CT-defined low skeletal muscle mass, who may have limited skeletal muscle reserve and greater vulnerability to adverse postoperative outcomes after major surgery (12, 13). More broadly, the advantages of laparoscopic surgery should be considered within an integrated perioperative strategy rather than attributed solely to a smaller incision. Contemporary laparoscopic practice emphasizes avoidance of unnecessary tissue injury, appropriate procedural simplification, effective postoperative analgesia, and early functional recovery. For example, previous studies have examined less extensive laparoscopic repair strategies for perforated peptic ulcer and compared regional analgesic techniques after laparoscopic cholecystectomy (14, 15). Although these studies involved different diseases and procedures and cannot be directly extrapolated to liver resection, they illustrate the broader principle that reducing procedural and analgesic burden may facilitate postoperative recovery. This principle may be especially important in patients with sarcopenia and limited physiological reserve.

More importantly, the favorable long-term associations of LLR were consistent across complementary analytic approaches. With median follow-up exceeding 4.5 years in both groups, LLR was associated with more favorable RFS and OS in the unweighted, PSM, ATO, and sIPTW analyses. The use of overlap weighting, which focuses on patients with greater clinical equipoise and reduces sensitivity to extreme propensity scores, provided an additional assessment of robustness in this observational setting (16, 17). Similar associations were also observed in expanded multivariable Cox models incorporating available tumor location, pathological, surgical, and treatment-related factors, including MVI, tumor differentiation, satellite nodules, surgical margin, and postoperative adjuvant therapy. Moreover, recurrence patterns, timing of the first RFS event, and post-recurrence treatment strategies were broadly comparable between groups in the PSM cohort, suggesting that the observed OS difference was not readily explained by marked differences in the type or subsequent management of recurrence. Clinically, these findings may reflect the cumulative consequences of a lower perioperative burden, including reduced blood loss and fewer postoperative complications, which could facilitate postoperative recovery and help maintain physiological reserve in patients with limited skeletal muscle reserve (18). However, these potential pathways were not directly assessed in the present study. In addition, the choice of surgical approach is influenced by proximity to major vascular structures, technical complexity, and surgeon experience (19, 20). Therefore, the present findings are best interpreted as robust observational associations that warrant prospective evaluation, rather than as evidence of a direct antitumor effect of laparoscopy.

The observed long-term associations are biologically plausible for a population with CT-defined sarcopenia but should be considered hypothesis-generating. Sarcopenia has repeatedly been linked to inferior survival outcomes in HCC, with updated meta-analytic evidence demonstrating worse overall survival and higher recurrence risk among sarcopenic patients (21). The prognostic relevance of CT-defined sarcopenia is not unique to HCC, as meta-analyses in colorectal and pancreatic cancer have similarly shown associations between low skeletal muscle mass and poorer survival outcomes after treatment (22, 23). These data support the concept that reduced muscle reserve reflects a systemic vulnerability that may interact with surgical stress and subsequent cancer control. Within liver surgery, emerging evidence suggests that laparoscopic hepatectomy may be associated with less postoperative skeletal muscle loss than open hepatectomy (11). However, the present study was not designed to directly test inflammatory, nutritional, metabolic, or muscle-preservation mechanisms. Therefore, the proposed link between reduced perioperative burden, postoperative muscle preservation, and long-term oncologic outcomes should be interpreted as a clinical hypothesis requiring prospective validation.

From a clinical and nutritional perspective, CT-defined low skeletal muscle mass may serve not only as a prognostic marker but also as a trigger for more comprehensive perioperative nutritional and functional assessment (24). Preoperative CT-based body composition assessment may complement conventional evaluations of liver function, tumor burden, and operative risk by identifying patients with reduced muscle reserve Importantly, selection of a minimally invasive surgical approach should be considered complementary to, rather than a substitute for, nutritional and functional optimization. Patients with CT-defined low skeletal muscle mass may benefit from multimodal strategies incorporating nutritional screening, individualized energy and protein provision, resistance exercise, prehabilitation, and early postoperative mobilization (25, 26). Future prospective studies should therefore assess whether LLR is associated not only with fewer complications but also with better preservation of skeletal muscle and earlier recovery of oral intake, physical activity, and functional status. The interaction observed between BMI category and OS should be interpreted cautiously. Because BMI does not distinguish skeletal muscle from adipose tissue, it may inadequately characterize clinically relevant body composition phenotypes (27). Future studies incorporating muscle attenuation, visceral adiposity, sarcopenic obesity, muscle strength, and physical performance may help determine whether specific body composition phenotypes modify the association between surgical approach and clinical outcomes.

Several limitations should be considered. First, this was a retrospective single-center study, and residual confounding and selection bias cannot be fully excluded despite DAG-informed covariate specification, propensity score matching, overlap weighting, and stabilized inverse probability weighting. Although additional tumor location, pathological, surgical, and treatment-related variables were incorporated into expanded Cox analyses where available, formal operative difficulty scores and surgeon-specific learning curve metrics were not routinely recorded. Second, sarcopenia was operationally defined using CT-derived L3 skeletal muscle index alone. This approach is objective, reproducible, and feasible in retrospective oncology cohorts, but it captures muscle quantity rather than muscle strength or physical performance, such as handgrip strength or gait speed. Therefore, some misclassification is possible, and the findings should be interpreted as applying to CT-defined sarcopenia rather than comprehensive consensus-defined sarcopenia. Third, perioperative inflammatory, nutritional, and metabolic biomarkers, as well as longitudinal postoperative skeletal muscle measurements, were not systematically available. Thus, we could not directly evaluate whether reduced surgical stress, improved nutritional recovery, or postoperative muscle preservation mediated the observed associations. Finally, the single-center, high-volume Chinese cohort with predominantly HBV-related HCC may limit generalizability to Western populations, non-HBV etiologies, and centers with less experience in laparoscopic liver resection.

In conclusion, among HCC patients with CT-defined sarcopenia, laparoscopic liver resection was associated with improved perioperative recovery and consistently favorable RFS and OS across matching, weighting, and expanded multivariable analyses, without apparent compromise in operative intent or technical oncologic quality indicators. Prospective multicenter studies incorporating standardized body composition assessment, functional sarcopenia measures, operative complexity reporting, and post-recurrence treatment data are needed to validate these findings and refine patient selection.

## Data Availability

The raw data supporting the conclusions of this article will be made available by the authors, without undue reservation.

## Supplementary material

SUPPLEMENTARY FIGURE S1. Directed acyclic graph for covariate selection. Simplified directed acyclic graph (DAG) depicting the assumed causal structure relating surgical approach (laparoscopic vs. open liver resection) to long-term outcomes (RFS/OS) in the study cohort. Baseline covariates were grouped into conceptual domains, including Demographics, Body composition, Liver status, Tumor burden, and Baseline labs/biomarkers, which were considered potential confounders affecting both surgical approach and outcomes.

SUPPLEMENTARY FIGURE S2. Subgroup analyses in the propensity score–matched cohort. Forest plots showing hazard ratios (HRs) and 95% confidence intervals for the association between surgical approach (laparoscopic liver resection vs. open liver resection) and **(A)** recurrence-free survival (RFS) and **(B)** overall survival (OS) across prespecified subgroups. AFP, alpha-fetoprotein; ALBI, albumin–bilirubin; BCLC, Barcelona Clinic Liver Cancer; BMI, body mass index; HBV, hepatitis B virus; HR, hazard ratio; OS, overall survival; RFS, recurrence-free survival.
