# Supplementary material for: Laparoscopic versus open liver resection in hepatocellular carcinoma patients with sarcopenia: a retrospective cohort study
Source: Front Nutr. 2026 Sep 30;13:1821779. doi: 10.3389/fnut.2026.1821779 (PMC13663880; doi:10.3389/fnut.2026.1821779)
Supplement: Supplementary file 1 [file Table_1.DOCX]

Table S1. Univariate and multivariate analysis of recurrence-free survival

| **Characteristic** |  |  | **Univariable** | | | **Multivariate** | | |
| --- | --- | --- | --- | --- | --- | --- | --- | --- |
|  | **N** | **Event N** | **HR** | **95% CI** | **p value** | **HR** | **95% CI** | **p value** |
| **Age** |  |  |  |  |  |  |  |  |
| < 65 | 233 | 135 | — | — |  | — | — |  |
| ≥ 65 | 52 | 24 | 0.73 | 0.47, 1.12 | 0.150 | 0.78 | 0.50, 1.23 | 0.285 |
| **Sex** |  |  |  |  |  |  |  |  |
| Female | 55 | 26 | — | — |  | — | — |  |
| Male | 230 | 133 | 1.14 | 0.75, 1.74 | 0.536 | 1.00 | 0.64, 1.55 | 0.992 |
| **BMI** |  |  |  |  |  |  |  |  |
| < 28 | 119 | 65 | — | — |  |  |  |  |
| ≥ 28 | 166 | 94 | 1.08 | 0.78, 1.48 | 0.650 |  |  |  |
| **Smoking history** |  |  |  |  |  |  |  |  |
| Non-current | 167 | 86 | — | — |  |  |  |  |
| Current | 118 | 73 | 1.35 | 0.99, 1.84 | 0.062 |  |  |  |
| **Alcohol consumption history** |  |  |  |  |  |  |  |  |
| Non-heavy | 182 | 95 | — | — |  |  |  |  |
| Heavy | 103 | 64 | 1.37 | 1.00, 1.89 | 0.051 |  |  |  |
| **Child-Pugh grade** |  |  |  |  |  |  |  |  |
| A | 281 | 155 | — | — |  | — | — |  |
| B | 4 | 4 | 2.79 | 1.03, 7.56 | 0.043 | 2.69 | 0.96, 7.52 | 0.060 |
| **HBV infection** |  |  |  |  |  |  |  |  |
| Without | 59 | 30 | — | — |  |  |  |  |
| With | 226 | 129 | 1.20 | 0.80, 1.78 | 0.375 |  |  |  |
| **WBC (*10^9/L)** |  |  |  |  |  |  |  |  |
| < 10 | 275 | 152 | — | — |  |  |  |  |
| ≥ 10 | 10 | 7 | 1.73 | 0.81, 3.69 | 0.157 |  |  |  |
| **Hb (g/L)** |  |  |  |  |  |  |  |  |
| ≥ 120 | 261 | 144 | — | — |  |  |  |  |
| < 120 | 24 | 15 | 1.28 | 0.75, 2.18 | 0.367 |  |  |  |
| **PLT (*10^9/L)** |  |  |  |  |  |  |  |  |
| ≥ 100 | 235 | 131 | — | — |  |  |  |  |
| < 100 | 50 | 28 | 0.89 | 0.59, 1.34 | 0.590 |  |  |  |
| **ALT (U/L)** |  |  |  |  |  |  |  |  |
| < 40 | 188 | 109 | — | — |  |  |  |  |
| ≥ 40 | 97 | 50 | 0.83 | 0.60, 1.17 | 0.291 |  |  |  |
| **AST (U/L)** |  |  |  |  |  |  |  |  |
| < 40 | 194 | 112 | — | — |  |  |  |  |
| ≥ 40 | 91 | 47 | 0.96 | 0.68, 1.36 | 0.830 |  |  |  |
| **GGT (U/L)** |  |  |  |  |  |  |  |  |
| < 50 | 152 | 74 | — | — |  | — | — |  |
| ≥ 50 | 133 | 85 | 1.58 | 1.16, 2.16 | 0.004 | 1.17 | 0.81, 1.67 | 0.399 |
| **ALP (U/L)** |  |  |  |  |  |  |  |  |
| < 130 | 256 | 133 | — | — |  | — | — |  |
| ≥ 130 | 29 | 26 | 2.29 | 1.50, 3.49 | <0.001 | 1.50 | 0.93, 2.41 | 0.093 |
| **HBV DNA (IU/mL)** |  |  |  |  |  |  |  |  |
| < 2000 | 182 | 97 | — | — |  |  |  |  |
| ≥ 2000 | 103 | 62 | 1.32 | 0.96, 1.82 | 0.086 |  |  |  |
| **AFP (ug/L)** |  |  |  |  |  |  |  |  |
| < 400 | 138 | 64 | — | — |  | — | — |  |
| ≥ 400 | 147 | 95 | 1.70 | 1.24, 2.33 | 0.001 | 1.35 | 0.97, 1.89 | 0.078 |
| **ALBI grade** |  |  |  |  |  |  |  |  |
| 1 | 217 | 116 | — | — |  |  |  |  |
| 2 | 68 | 43 | 1.34 | 0.94, 1.90 | 0.105 |  |  |  |
| **Tumor location** |  |  |  |  |  |  |  |  |
| Peripheral | 168 | 101 | — | — |  |  |  |  |
| Central | 117 | 58 | 0.76 | 0.55, 1.05 | 0.098 |  |  |  |
| **Maximum tumor diameter (cm)** |  |  |  |  |  |  |  |  |
| < 5 | 128 | 59 | — | — |  | — | — |  |
| ≥ 5 | 157 | 100 | 1.85 | 1.34, 2.56 | <0.001 | 1.43 | 0.98, 2.10 | 0.063 |
| **Tumor number** |  |  |  |  |  |  |  |  |
| Single | 277 | 153 | — | — |  |  |  |  |
| Multiple | 8 | 6 | 1.70 | 0.75, 3.84 | 0.205 |  |  |  |
| **Cirrhosis** |  |  |  |  |  |  |  |  |
| Without | 102 | 56 | — | — |  |  |  |  |
| With | 183 | 103 | 1.06 | 0.77, 1.47 | 0.723 |  |  |  |
| **MVI** |  |  |  |  |  |  |  |  |
| Without | 135 | 65 | — | — |  | — | — |  |
| With | 150 | 94 | 1.61 | 1.18, 2.22 | 0.003 | 1.18 | 0.81, 1.71 | 0.381 |
| **Differentiation** |  |  |  |  |  |  |  |  |
| I–II | 147 | 86 | — | — |  |  |  |  |
| III–IV | 138 | 73 | 0.81 | 0.59, 1.11 | 0.189 |  |  |  |
| **Satellite** |  |  |  |  |  |  |  |  |
| Without | 234 | 126 | — | — |  |  |  |  |
| With | 51 | 33 | 1.27 | 0.86, 1.86 | 0.229 |  |  |  |
| **Surgical approach** |  |  |  |  |  |  |  |  |
| OLR | 175 | 111 | — | — |  | — | — |  |
| LLR | 110 | 48 | 0.57 | 0.41, 0.81 | 0.001 | 0.63 | 0.44, 0.88 | 0.008 |
| **Surgical resection type** |  |  |  |  |  |  |  |  |
| Non-anatomical resection | 231 | 125 | — | — |  |  |  |  |
| Anatomical resection | 54 | 34 | 1.21 | 0.82, 1.76 | 0.334 |  |  |  |
| **Surgical margin (cm)** |  |  |  |  |  |  |  |  |
| < 1 | 121 | 76 | — | — |  | — | — |  |
| ≥ 1 | 164 | 83 | 0.71 | 0.52, 0.97 | 0.031 | 0.80 | 0.58, 1.10 | 0.171 |
| **Adjuvant therapy** |  |  |  |  |  |  |  |  |
| Without | 226 | 123 | — | — |  |  |  |  |
| With | 59 | 36 | 1.27 | 0.87, 1.84 | 0.215 |  |  |  |

**Abbreviations:** AFP, alpha-fetoprotein; ALBI, albumin–bilirubin; ALP, alkaline phosphatase; ALT, alanine aminotransferase; AST, aspartate aminotransferase; BCLC, Barcelona Clinic Liver Cancer; BMI, body-mass index; GGT, gamma-glutamyl transferase; Hb, haemoglobin; HBV, hepatitis B virus; LLR laparoscopic liver resection; OLR open liver resection; PLT, platelet count; WBC, white blood cell count.
