# Supplementary material for: Laparoscopic versus open liver resection in hepatocellular carcinoma patients with sarcopenia: a retrospective cohort study
Source: Front Nutr. 2026 Sep 30;13:1821779. doi: 10.3389/fnut.2026.1821779 (PMC13663880; doi:10.3389/fnut.2026.1821779)
Supplement: Supplementary file 2 [file Table_2.DOCX]

Table S2. Univariate and multivariate analysis of overall survival

| **Characteristic** |  |  | **Univariable** | | | **Multivariate** | | |
| --- | --- | --- | --- | --- | --- | --- | --- | --- |
|  | **N** | **Event N** | **HR** | **95% CI** | **p value** | **HR** | **95% CI** | **p value** |
| **Age** |  |  |  |  |  |  |  |  |
| < 65 | 233 | 104 | — | — |  | — | — |  |
| ≥ 65 | 52 | 20 | 0.79 | 0.49, 1.28 | 0.338 | 0.85 | 0.52, 1.39 | 0.524 |
| **Sex** |  |  |  |  |  |  |  |  |
| Female | 55 | 22 | — | — |  | — | — |  |
| Male | 230 | 102 | 1.04 | 0.65, 1.65 | 0.873 | 0.85 | 0.51, 1.42 | 0.540 |
| **BMI** |  |  |  |  |  |  |  |  |
| < 28 | 119 | 53 | — | — |  |  |  |  |
| ≥ 28 | 166 | 71 | 0.99 | 0.69, 1.42 | 0.963 |  |  |  |
| **Smoking history** |  |  |  |  |  |  |  |  |
| Non-current | 167 | 68 | — | — |  |  |  |  |
| Current | 118 | 56 | 1.33 | 0.93, 1.89 | 0.118 |  |  |  |
| **Alcohol consumption history** |  |  |  |  |  |  |  |  |
| Non-heavy | 182 | 72 | — | — |  | — | — |  |
| Heavy | 103 | 52 | 1.46 | 1.02, 2.08 | 0.039 | 1.48 | 1.00, 2.20 | 0.050 |
| **Child-Pugh grade** |  |  |  |  |  |  |  |  |
| A | 281 | 121 | — | — |  |  |  |  |
| B | 4 | 3 | 1.58 | 0.50, 4.97 | 0.437 |  |  |  |
| **HBV infection** |  |  |  |  |  |  |  |  |
| Without | 59 | 25 | — | — |  |  |  |  |
| With | 226 | 99 | 1.16 | 0.75, 1.80 | 0.507 |  |  |  |
| **WBC (*10^9/L)** |  |  |  |  |  |  |  |  |
| < 10 | 275 | 119 | — | — |  |  |  |  |
| ≥ 10 | 10 | 5 | 1.17 | 0.48, 2.87 | 0.727 |  |  |  |
| **Hb (g/L)** |  |  |  |  |  |  |  |  |
| ≥ 120 | 261 | 113 | — | — |  |  |  |  |
| < 120 | 24 | 11 | 1.21 | 0.65, 2.24 | 0.554 |  |  |  |
| **PLT (*10^9/L)** |  |  |  |  |  |  |  |  |
| ≥ 100 | 235 | 104 | — | — |  |  |  |  |
| < 100 | 50 | 20 | 0.77 | 0.48, 1.24 | 0.287 |  |  |  |
| **ALT (U/L)** |  |  |  |  |  |  |  |  |
| < 40 | 188 | 80 | — | — |  |  |  |  |
| ≥ 40 | 97 | 44 | 0.95 | 0.65, 1.37 | 0.769 |  |  |  |
| **AST (U/L)** |  |  |  |  |  |  |  |  |
| < 40 | 194 | 80 | — | — |  |  |  |  |
| ≥ 40 | 91 | 44 | 1.25 | 0.86, 1.80 | 0.244 |  |  |  |
| **GGT (U/L)** |  |  |  |  |  |  |  |  |
| < 50 | 152 | 57 | — | — |  | — | — |  |
| ≥ 50 | 133 | 67 | 1.53 | 1.07, 2.18 | 0.018 | 0.95 | 0.63, 1.43 | 0.789 |
| **ALP (U/L)** |  |  |  |  |  |  |  |  |
| < 130 | 256 | 99 | — | — |  | — | — |  |
| ≥ 130 | 29 | 25 | 2.88 | 1.86, 4.48 | <0.001 | 1.70 | 0.99, 2.91 | 0.054 |
| **HBV DNA (IU/mL)** |  |  |  |  |  |  |  |  |
| < 2000 | 182 | 68 | — | — |  | — | — |  |
| ≥ 2000 | 103 | 56 | 1.64 | 1.15, 2.34 | 0.006 | 1.36 | 0.93, 1.98 | 0.114 |
| **AFP (ug/L)** |  |  |  |  |  |  |  |  |
| < 400 | 138 | 44 | — | — |  | — | — |  |
| ≥ 400 | 147 | 80 | 1.98 | 1.37, 2.85 | <0.001 | 1.60 | 1.08, 2.37 | 0.020 |
| **ALBI grade** |  |  |  |  |  |  |  |  |
| 1 | 217 | 88 | — | — |  |  |  |  |
| 2 | 68 | 36 | 1.47 | 1.00, 2.16 | 0.053 |  |  |  |
| **Tumor location** |  |  |  |  |  |  |  |  |
| Peripheral | 168 | 79 | — | — |  |  |  |  |
| Central | 117 | 45 | 0.76 | 0.53, 1.10 | 0.143 |  |  |  |
| **Maximum tumor diameter (cm)** |  |  |  |  |  |  |  |  |
| < 5 | 128 | 38 | — | — |  | — | — |  |
| ≥ 5 | 157 | 86 | 2.36 | 1.61, 3.46 | <0.001 | 1.87 | 1.20, 2.92 | 0.006 |
| **Tumor number** |  |  |  |  |  |  |  |  |
| Single | 277 | 119 | — | — |  |  |  |  |
| Multiple | 8 | 5 | 1.39 | 0.57, 3.40 | 0.470 |  |  |  |
| **Cirrhosis** |  |  |  |  |  |  |  |  |
| Without | 102 | 43 | — | — |  |  |  |  |
| With | 183 | 81 | 1.08 | 0.75, 1.57 | 0.666 |  |  |  |
| **MVI** |  |  |  |  |  |  |  |  |
| Without | 135 | 47 | — | — |  | — | — |  |
| With | 150 | 77 | 1.87 | 1.30, 2.70 | <0.001 | 1.22 | 0.79, 1.89 | 0.367 |
| **Differentiation** |  |  |  |  |  |  |  |  |
| I–II | 147 | 64 | — | — |  |  |  |  |
| III–IV | 138 | 60 | 0.92 | 0.65, 1.31 | 0.650 |  |  |  |
| **Satellite** |  |  |  |  |  |  |  |  |
| Without | 234 | 99 | — | — |  |  |  |  |
| With | 51 | 25 | 1.17 | 0.76, 1.82 | 0.476 |  |  |  |
| **Surgical approach** |  |  |  |  |  |  |  |  |
| OLR | 175 | 90 | — | — |  | — | — |  |
| LLR | 110 | 34 | 0.52 | 0.35, 0.78 | 0.001 | 0.62 | 0.41, 0.93 | 0.021 |
| **Surgical resection type** |  |  |  |  |  |  |  |  |
| Non-anatomical resection | 231 | 98 | — | — |  |  |  |  |
| Anatomical resection | 54 | 26 | 1.13 | 0.73, 1.74 | 0.584 |  |  |  |
| **Surgical margin (cm)** |  |  |  |  |  |  |  |  |
| < 1 | 121 | 62 | — | — |  |  |  |  |
| ≥ 1 | 164 | 62 | 0.70 | 0.50, 1.00 | 0.051 |  |  |  |
| **Adjuvant therapy** |  |  |  |  |  |  |  |  |
| Without | 226 | 93 | — | — |  |  |  |  |
| With | 59 | 31 | 1.41 | 0.93, 2.11 | 0.102 |  |  |  |

**Abbreviations:** AFP, alpha-fetoprotein; ALBI, albumin–bilirubin; ALP, alkaline phosphatase; ALT, alanine aminotransferase; AST, aspartate aminotransferase; BCLC, Barcelona Clinic Liver Cancer; BMI, body-mass index; GGT, gamma-glutamyl transferase; Hb, haemoglobin; HBV, hepatitis B virus; LLR laparoscopic liver resection; OLR open liver resection; PLT, platelet count; WBC, white blood cell count.
