# Supplementary material for: Laparoscopic versus open liver resection in hepatocellular carcinoma patients with sarcopenia: a retrospective cohort study
Source: Front Nutr. 2026 Sep 30;13:1821779. doi: 10.3389/fnut.2026.1821779 (PMC13663880; doi:10.3389/fnut.2026.1821779)
Supplement: Supplementary file 3 [file Table_3.DOCX]

Supplementary Table 3. Recurrence patterns and post-recurrence treatment strategies in the propensity score-matched cohort

|  | LLR  (n=41/96) | OLR  (n=58/96) | p value |
| --- | --- | --- | --- |
| **Type of first RFS event, n (%)** |  |  | 0.681 |
| Intrahepatic recurrence | 31 (75.6%) | 43 (74.1%) |  |
| Extrahepatic recurrence | 3 (7.3%) | 3 (5.2%) |  |
| Intrahepatic and extrahepatic recurrence | 6 (14.6%) | 7 (12.1%) |  |
| Death | 1 (2.4%) | 5 (8.6%) |  |
| **Time to first RFS event, n (%)** |  |  | 0.857 |
| Early recurrence ≤2 years | 24 (58.5%) | 35 (60.3%) |  |
| Late recurrence >2 years | 17 (41.5%) | 23 (39.7%) |  |
| **Post-recurrence treatment*, n (%)** |  |  | 0.778 |
| Repeat resection | 14 (35.0%) | 16 (30.2%) |  |
| Ablation | 7 (17.5%) | 15 (28.3%) |  |
| TACE/HAIC | 9 (22.5%) | 12 (22.6%) |  |
| Systemic therapy | 8 (20.0%) | 9 (17.0%) |  |
| Radiotherapy | 1 (2.5%) | 1 (1.9%) |  |
| Best supportive care | 1 (2.5%) | 0 (0.0%) |  |

*Post-recurrence treatment strategies were calculated among patients with documented recurrence and are presented as percentages of recurrent cases in each group.

**Abbreviations:** LLR, laparoscopic liver resection; OLR, open liver resection; RFS, recurrence-free survival; TACE, transarterial chemoembolization; HAIC, hepatic arterial infusion chemotherapy.
